# Pharmacokinetic Assessment of Atazanavir and Favipiravir Following *Echinacea* Supplementation: A Controlled Herb–Drug Interaction Investigation

**DOI:** 10.1002/bdd.70034

**Published:** 2026-05-30

**Authors:** Siva Nageswara Rao Gajula, Jeevan Sai Kallam, Aarika Kanukolanu, Ganesh Korada, Lakshmi Vineela Nalla

**Affiliations:** ^1^ Department of Pharmaceutical Analysis GITAM School of Pharmacy GITAM (Deemed to be University) Visakhapatnam Andhra Pradesh India; ^2^ Multidisciplinary Unit of Research on Translational Initiatives‐Sophisticated Analytical Instrumentation Facility (MURTI‐SAIF) GITAM (Deemed to be University) Visakhapatnam Andhra Pradesh India; ^3^ Department of Pharmacology GITAM School of Pharmacy GITAM (Deemed to be University) Visakhapatnam Andhra Pradesh India

**Keywords:** antiviral therapy, atazanavir, *Echinacea*, favipiravir, herb‐drug interaction, LC‐MS/MS, pharmacokinetics

## Abstract

*Echinacea* is a widely used herbal supplement and is frequently co‐administered with antiviral drugs, raising concerns regarding potential herb–drug interactions. This study evaluated the effect of repeated *Echinacea* administration on the pharmacokinetics of favipiravir and atazanavir in rats. Male Sprague Dawley rats were administered *Echinacea* (250 mg/kg) or vehicle once daily for 10 days, followed by a single oral dose of favipiravir (70 mg/kg) or atazanavir (35 mg/kg). Plasma drug concentrations were quantified using validated LC–MS/MS methods, and pharmacokinetic parameters were determined by noncompartmental analysis. *Echinacea* co‐administration did not significantly alter favipiravir exposure (*C*_max_: 39,677 ± 5385 vs. 36,364 ± 5379 ng/mL; AUC0–t: 144,457 ± 17,267 vs. 104,840 ± 27,001 ng h/mL) or atazanavir exposure (*C*_max_: 3274 ± 256 vs. 4004 ± 629 ng/mL; AUC0–t: 8962 ± 1667 vs. 10,645 ± 2949 ng h/mL). No relevant changes were observed in clearance, half‐life, or mean residence time for either drug. These findings indicate that repeated *Echinacea* administration does not result in a pharmacokinetically relevant interaction with favipiravir or atazanavir under the investigated conditions.

## Graphical abstract

Pharmacokinetic interactions between *Echinacea* and two antiviral drugs, favipiravir and atazanavir, were assessed in rats. The findings suggest that *Echinacea* does not significantly affect the pharmacokinetics of favipiravir and atazanavir. These results provide preliminary evidence that concurrent use of *Echinacea* with these antiviral drugs may be safe.

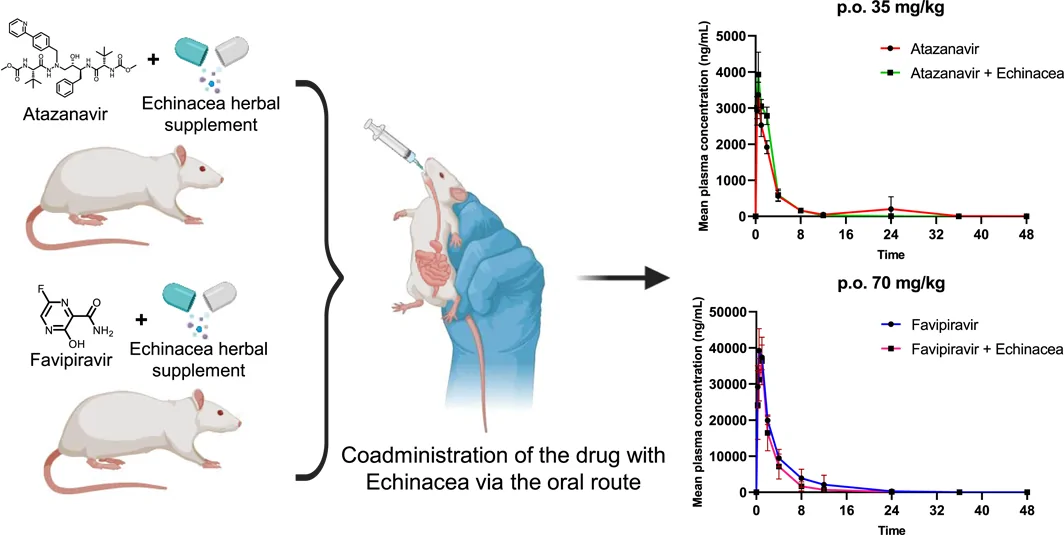

## 1 Introduction

The concurrent use of herbal supplements and conventional pharmaceutical medications has become increasingly prevalent globally, driven by a growing interest in natural remedies and the accessibility of over‐the‐counter herbal products (Eichhorn et al. 2011). Although many individuals perceive herbal supplements as safe and benign, the potential for significant herb–drug interactions (HDIs) poses a considerable clinical concern (Dores et al. 2023). These interactions can lead to unpredictable alterations in the pharmacokinetic and pharmacodynamic profiles of both herbal and synthetic drugs (Rao Gajula et al. 2021, 2020). Therefore, HDIs can result in sub‐therapeutic drug concentrations, leading to treatment failure and the development of drug resistance, or conversely, elevated drug levels, increasing the risk of adverse drug reactions and toxicities (Fasinu et al. 2012). Hence, a comprehensive understanding of HDIs' potential is paramount for healthcare professionals to ensure medication safety, optimize therapeutic outcomes, and provide informed guidance to patients.

*Echinacea*, a popular herbal supplement derived from various species of the *Echinacea* genus, is widely marketed and consumed for its purported immunostimulatory (El‐Ashmawy et al. 2015), anti‐inflammatory (Merali et al. 2003), and antiviral properties (Hudson and Vimalanathan 2011). It is frequently used as a self‐medication strategy for the prevention and management of common cold and flu symptoms, as well as for general immune system support (Mammari et al. 2023). The complex phytochemical composition of *Echinacea* encompasses a diverse array of bioactive compounds, including alkylamides, polysaccharides, caffeic acid derivatives, and flavonoids (Ahmadi et al. 2024). These constituents are believed to contribute, individually or synergistically, to the herb's observed pharmacological effects, although the precise mechanisms of action for all components remain under investigation.

Atazanavir, a second‐generation azapeptide HIV‐1 protease inhibitor, plays a vital role in combination antiretroviral therapy for the management of human immunodeficiency virus (HIV) infection (Voshavar 2019). By selectively inhibiting the viral protease enzyme, atazanavir prevents the cleavage of viral polyproteins, thereby blocking the maturation of infectious viral particles (Voshavar 2019). However, the pharmacokinetic profile of atazanavir is characterized by significant inter‐individual variability and susceptibility to drug interactions (Montanha et al. 2022). Atazanavir is primarily metabolized by the cytochrome P450 (CYP) enzyme system, with CYP3A4 being the major isoform involved in its oxidative metabolism (Orrick and Steinhart 2004). Furthermore, atazanavir acts as both a substrate and an inhibitor of P‐glycoprotein (P‐gp). This efflux transporter protein plays a crucial role in the absorption, distribution, and elimination of drugs (Perloff et al. 2005). Consequently, co‐administration of substances that modulate CYP3A4 or P‐gp activity can significantly alter ATV plasma concentrations, potentially leading to sub‐optimal virologic control, the development of drug resistance, or increased risk of adverse effects such as hyperbilirubinemia and cardiovascular complications.

Favipiravir, a pyrazine analog, is a broad‐spectrum antiviral drug that has demonstrated efficacy against various RNA viruses, including influenza viruses and, notably, certain emerging viral pathogens (Konstantinova et al. 2022). Its primary mechanism of action involves inhibiting the viral RNA‐dependent RNA polymerase (RdRp), an enzyme essential for viral genome replication (Peng et al. 2021). Favipiravir undergoes intracellular activation to its active form, favipiravir‐ribofuranosyl‐5′‐triphosphate (favipiravir‐RTP), which acts as a substrate for the RNA‐dependent RNA polymerase (RdRp), leading to chain termination during viral RNA synthesis (Hadj Hassine et al. 2022). The primary metabolic pathways of favipiravir involve hydrolysis to an inactive metabolite and oxidation by aldehyde oxidase (Hoseininezhad‐Namin et al. 2024). While CYP enzymes are not the primary metabolizers of FPV, potential interactions involving other metabolic enzymes or drug transporters cannot be entirely discounted, particularly with co‐administered agents that may influence these pathways or affect systemic drug exposure.

Given the widespread availability and use of *Echinacea* as a self‐care remedy and the therapeutic roles of atazanavir and favipiravir, the potential for pharmacokinetic interactions when used concomitantly warrants careful investigation. While existing literature provides some insights into the in vitro and in vivo effects of *Echinacea* extracts and their constituents on various CYP enzymes and P‐gp, specific data regarding the interaction potential between *Echinacea* and atazanavir, as well as between *Echinacea* and favipiravir, remain unexplored. Understanding these potential interactions is of significant clinical importance for healthcare providers managing patients who may be using these agents concurrently, particularly considering the potential for altered antiviral efficacy or increased toxicity. This research systematically investigated the in vitro interaction potential of a commercially available *Echinacea* supplement with the pharmacokinetic profiles of atazanavir and favipiravir. This study aims to provide valuable preclinical insights into the safety and efficacy of their co‐administration. The findings of this investigation will contribute to a more comprehensive understanding of potential HDIs involving *Echinacea* and these important antiviral drugs, thereby informing clinical decision‐making, optimizing patient management strategies, and ultimately enhancing patient safety and therapeutic outcomes.

## 2 Materials and Methods

### 2.1 Chemicals and Reagents

Atazanavir (purity ≥ 99%) and favipiravir (purity ≥ 99%) were received as a gift sample from Hetero Labs (Hyderabad, India). Ammonium formate (LC–MS grade), formic acid (LC‐MS grade), methanol (LC‐MS grade), acetonitrile (LC‐MS grade), sodium carboxymethyl cellulose, and disodium EDTA dihydrate were sourced from Sigma‐Aldrich (Bangalore, India). Water used in the study was obtained from Milli‐Q Type 1 ultrapure water systems. The *Echinacea* supplement used in this study was obtained from Biotrex Nutraceuticals, a commercially available preparation containing 400 mg of *Echinacea* purpurea extract (aerial parts), standardized to 4% total polyphenols, as claimed by the manufacturer. The product was obtained from Biotrex Nutraceuticals. All doses were prepared from the same production batch (Bath No. OBC175) to ensure consistency across the study.

### 2.2 Instrumentation and Conditions

The analytical procedure used liquid chromatography–tandem mass spectrometry (LC–MS/MS), comprising an AB SCEX Exion LC system coupled to a QTRAP 5500+ mass spectrometer. Mass detection was performed by electrospray ionization in positive ion mode. Internal calibration of the mass spectrometer was performed using a solution with a wide mass range. The optimized mass spectrometry conditions of atazanavir, favipiravir, ziprasidone, and tolbutamide are given in Table 1. All data were acquired under consistent instrumental parameters and processed using Analyst software (Version 1.7.3). Chromatographic separation was achieved on a Phenomenex C18 column (150 × 3 mm, 2.6 μm, 100 Å). The mobile phase consisted of ammonium formate in water (pH 3.0) and methanol, with an isocratic elution of 20:80 (water: methanol) and a run time of 4 min for the separation and estimation of atazanavir and ziprasidone. On the other hand, the mobile phase consisted of 0.2% formic acid in water (Solvent A) and acetonitrile (Solvent B). A gradient elution program was employed with the following time (min)/%ACN profile: 0.0–0.0/20, 0.0–1.0/20, 1.0–3.0/80, 3.0–4.0/80, 4.0–5.0/20, and 5.0–7.0/20 for the separation and estimation of favipiravir and tolbutamide. For both methods, the flow rate was maintained at 0.3 mL/min, the column temperature was set to 40°C, and the autosampler was maintained at 5°C. Each injection had a volume of 5 μL.

**TABLE 1:** Optimized LC–MS/MS parameters for analytes and internal standards.

Analyte	MRM transition	Ion spray voltage	Source temperature	Nebulizer gas	Decluttering potential	Collision energy	Entrance potential
Atazanavir	705.3 m/z to 335.2 m/z	5500 V	500°C	45 psi	100 V	30 V	10 V
Ziprasidone	413.1 m/z to 194.0 m/z	5500V	500°C	45 psi	100 V	30 V	10 V
Favipiravir	156.1 m/z to 113.1 m/z	−4500 V	500°C	50 psi	−100 V	−22 V	−10 V
Tolbutamide	269.0 m/z to 170 m/z	−4500 V	500°C	50 psi	−80 V	−25 V	−10 V

*Note:* Multiple reaction monitoring (MRM) transitions, ionization conditions, and source parameters are listed for atazanavir, favipiravir, and their respective internal standards (ziprasidone and tolbutamide).

### 2.3 Preparation of Stock and Calibration Standards for Atazanavir and Favipiravir

#### 2.3.1 Stock Solutions

Stock solutions of atazanavir and favipiravir were initially prepared in methanol at 1000 μg/mL. These were further diluted with the same solvent to obtain working standard solutions at 20 μg/mL. Likewise, the internal standards (ziprasidone and tolbutamide) were prepared in methanol at 1000 μg/mL and subsequently diluted to 100 ng/mL for analytical use.

#### 2.3.2 Calibration Standards

To establish calibration curves, appropriate volumes of the atazanavir working solution were spiked into drug‐free rat plasma to achieve the following seven calibration levels: 10, 25, 50, 100, 250, 500, and 1000 ng/mL. The same procedure was followed for favipiravir to prepare matching concentration standards (10, 25, 50, 100, 250, 500, 1000, and 2000 ng/mL). In addition, quality control (QC) samples at three concentration levels were prepared independently: low QC (LQC) at 30 ng/mL, medium QC (MQC) at 800 ng/mL, and high QC (HQC) at 1600 ng/mL.

### 2.4 Sample Preparation

Plasma samples were processed by precipitating proteins with ice‐cold methanol (precooled to −20°C) containing the respective analyte IS (100 ng/mL). After adding this crashing solvent, the samples were vortexed for 10 min, followed by centrifugation at 10,000 rpm for 10 min at 4°C. The resulting supernatant was carefully transferred to autosampler vials for LC‐QTRAP/MS analysis using the optimized method.

### 2.5 Method Validation

The LC‐QTRAP/MS methods developed for analyzing atazanavir and favipiravir were validated according to the guidelines outlined in the U.S. Food and Drug Administration (FDA) “Bioanalytical Method Validation” [19] and the European Medicines Agency (EMA) guidelines for validating bioanalytical methods (EMA 2011).

#### 2.5.1 Selectivity and Carryover

Selectivity was evaluated by analyzing blank plasma samples from six different sources to ensure that no interference from endogenous substances or other compounds occurred. Carryover was assessed by injecting the upper limit of quantification (ULOQ) samples, followed by blank injections, with a defined injection sequence ensuring no more than 20% of the lower limit of quantification (LLOQ) signal in blank samples.

#### 2.5.2 Recovery and Matrix Effect

Recovery of atazanavir and favipiravir from rat plasma was determined by comparing peak areas of pre‐extracted and post‐extracted samples at LQC, MQC, and HQC concentrations. The percentage recovery was determined using the following formula (Gajula et al. 2025, 2024). The analyte's recovery must remain consistent and reproducible throughout the analysis.

$$\%\;\text{Recovery}=\frac{\text{Response}\;\text{of}\;\text{pre‐}\text{extracted}\;\text{sample}}{\text{Response}\;\text{of}\;\text{post‐}\text{extracted}\;\text{sample}}\times 100$$

Matrix effects were evaluated using the post‐extraction spike method, ensuring the analyte's response in the presence of matrix components. This approach entails comparing the analyte's response in a pure solution (absent matrix components) with that in a post‐extraction spiked sample, which includes both the analyte and the biological matrix. The percentage matrix effect should be ± 15%. The percentage matrix effect can be calculated using the following formula (Gajula et al. 2025, 2024):

$$\%\;\text{Matrix}\;\text{effect}=\left(\frac{\text{Response}\;\text{of}\;\text{post‐}\text{extracted}\;\text{sample}}{\text{Response}\;\text{of}\;\text{neat}\;\text{analyte}\;\text{solution}}-1\right)\times 100$$

#### 2.5.3 Linearity and Sensitivity

Linearity was established over a concentration range of 10–2000 ng/mL for both atazanavir and favipiravir in rat plasma using a weighted 1/x quadratic regression model, with a correlation coefficient (*r* ^2^) ≥ 0.99. Sensitivity was confirmed by achieving a signal‐to‐noise ratio (S/N) ≥ 10:1 at the lower limit of quantification (LLOQ) of 10 ng/mL.

#### 2.5.4 Precision and Accuracy

Intraday and inter‐day precision, expressed as relative standard deviation (%RSD), and accuracy, expressed as relative error (%RE), were assessed by analyzing LLOQ and QC samples in rat plasma. Precision and accuracy criteria were met within ± 15% (± 20% at LLOQ) of nominal concentrations.

#### 2.5.5 Stability

The stability of atazanavir and favipiravir in rat plasma was evaluated under various storage conditions, including benchtop, long‐term, autosampler, and freeze–thaw stability. Samples demonstrated stability with precision and accuracy within acceptable limits across the specified conditions.

#### 2.5.6 Dilution Integrity and Incurred Sample Reanalysis (ISR)

Plasma samples with analyte concentrations above the upper limit of quantification were diluted 20‐fold with blank rat plasma and reanalyzed within the validated calibration range. Dilution integrity was assessed at this dilution factor using quality control samples, with acceptance criteria of ± 15% for accuracy and ≤ 15% for precision in accordance with FDA and EMA bioanalytical guidelines. Incurred sample reanalysis (ISR) was performed for 10% of study samples, including samples near *C*_max_ and in the elimination phase. Reanalyzed concentrations were within ± 20% of original values, confirming assay reproducibility.

### 2.6 Care and Maintenance of Animals

Sprague Dawley (SD) rats were procured from Vivo Bio Tech Ltd., Hyderabad, India. All animal studies were conducted with prior approval from the Institutional Animal Ethics Committee (IAEC) of GITAM, Visakhapatnam, India (Approval No. IAEC/GU‐1287/SNR‐F/7/August 2024), in accordance with the guidelines set by the Committee for the Control and Supervision of Experiments on Animals (CCSEA), Government of India. The animals were acclimated to laboratory conditions for 2 weeks before the experiments began. Throughout the acclimatization and study periods, the animals were housed in groups of three per cage with unrestricted access to food and water. The housing facility was CCSEA‐approved (Registration No. 1287/PO/Re/S/2009/CPCSEA) and maintained a 12‐h light/dark cycle, a controlled temperature of 25 ± 2°C, and a relative humidity range of 50%–60%. All procedures involving animals were conducted in accordance with institutionally approved protocols for the care and use of animals. Serial blood sampling was performed in compliance with CCSEA and IAEC guidelines. In adult rats (250–300 g), total blood volume is approximately 16–20 mL/kg; therefore, the cumulative volume collected (∼2.2 mL over 48 h) represented < 10% of circulating blood volume, within accepted limits for serial pharmacokinetic studies. All sampling intervals and volumes were pre‐approved in the IAEC protocol and complied with CCSEA guidelines for serial pharmacokinetic blood collection. Retro‐orbital sampling was conducted under isoflurane anesthesia to minimize pain and distress. Animals were monitored throughout the study for clinical signs, including lethargy, weight loss, bleeding, or ocular injury. No animals showed humane end points during the study, and no excessive bleeding or abnormal behavior was observed.

### 2.7 Experimental Design

To evaluate the effect of herb–drug interactions between an herb (*Echinacea* supplement) and drugs (atazanavir and favipiravir) in healthy rats, 24 SD rats were randomly assigned into four groups, each comprising six animals. Following an overnight fast, Group I and II rats received an oral dose of atazanavir (35 mg/kg) and favipiravir (70 mg/kg), respectively, prepared in 0.5% sodium CMC, once daily for 10 consecutive days. Group III and IV rats received the same antiviral dose together with *Echinacea* (250 mg/kg) once daily for 10 days. The doses of atazanavir and favipiravir were derived from their respective human therapeutic doses using body surface area–based allometric dose conversion for rats (animal equivalent dose, AED (mg/kg) = human dose (mg/kg) × 6.2), in accordance with standard interspecies scaling principles (Nair and Jacob 2016). *Echinacea* was administered at a dose of 250 mg/kg, consistent with doses used in a previous study (Mohamed et al. 2023). The *Echinacea* preparation used was supplied with manufacturer‐supplied standardization certificates, ensuring quality control; however, no further phytochemical standardization was conducted in this study. On Day 10, blood samples (∼0.2 mL) were collected from the retro‐orbital plexus into tubes containing disodium EDTA (pH 7.4) at predetermined time points: 0 (before dosing), 0.25, 0.5, 1, 1.5, 2, 4, 8, 12, 24, and 48 h post‐dose. All samples were promptly centrifuged at 10,000 rpm for 10 min at 4°C, and the separated plasma was stored at −80°C until further analysis.

### 2.8 Pharmacokinetics Analysis

The plasma concentration‐time data were utilized to determine the pharmacokinetic parameters of atazanavir and favipiravir. Noncompartmental analysis was conducted to calculate key parameters, including the maximum plasma concentration (*C*_max_), time to reach *C*_max_ (*T*_max_), terminal elimination half‐life (*t*_1/2_), mean residence time (MRT), apparent volume of distribution (Vz/F), apparent oral clearance (CL/F), and the area under the concentration‐time curve (AUC_0–t_ and AUC_0–∞_). All calculations were carried out using Phoenix WinNonlin software (Version 8.3, Pharsight Inc., USA).

### 2.9 Statistical Analysis

Data were presented as mean ± standard deviation (SD). For comparisons between two groups, unpaired two‐tailed *t*‐tests were applied as the data were normally distributed. All analyses were conducted using GraphPad Prism software (Version 8.0.2). Statistical significance was set at *p* < 0.05. In addition, a prospective power analysis was performed based on reported inter‐animal variability in the pharmacokinetics of atazanavir and favipiravir. Using a two‐group comparison (*α* = 0.05), a sample size of six animals per group provides ≥ 80% power to detect a ≥ 25% difference in AUC or *C*_max_, which is considered pharmacokinetically and regulatorily relevant for assessing drug interactions.

## 3 Results and Discussion

### 3.1 Method Development and Optimization

Method development for LC–MS/MS analysis typically begins with identifying the optimal mass spectrometric (MS) conditions to ensure reliable detection of the target analytes (Gajula et al. 2020, 2023; Rao Gajula et al. 2023). This includes evaluating whether the compounds can be detected without derivatization, selecting the most suitable ionization technique and determining the ionization polarity that provides the best sensitivity. Considering the chemical structures and ionizable groups of atazanavir, favipiravir, and the internal standards (ziprasidone and tolbutamide), MS conditions were optimized in positive ion mode (atazanavir and ziprasidone) and negative ion mode (favipiravir and tolbutamide) to achieve a stable and highly sensitive response with minimal SRM cross talk, without the need for derivatization. Both atazanavir and favipiravir demonstrated optimal sensitivity under electrospray ionization (ESI) with positive polarity for atazanavir and negative polarity for favipiravir, respectively. The use of an internal standard is a well‐established practice in LC–MS–based bioanalysis to minimize variability. Ziprasidone and tobutamide were chosen as the IS due to their physicochemical properties and ionization behavior being similar to those of atazanavir and favipiravir, respectively. Direct injections of the analytes and IS into the ESI source were performed, followed by detection in SRM mode. Optimization of the MS/MS parameters, carried out using Analyst software (Version 1.7.3), yielded ideal settings as listed in Table 1. Product ion scans confirmed that the selected SRM transitions showed minimal interference from other components. The product ion spectra for atazanavir, favipiravir, ziprasidone, and tolbutamide are shown in Figures 1 and 2.

**FIGURE 1:**
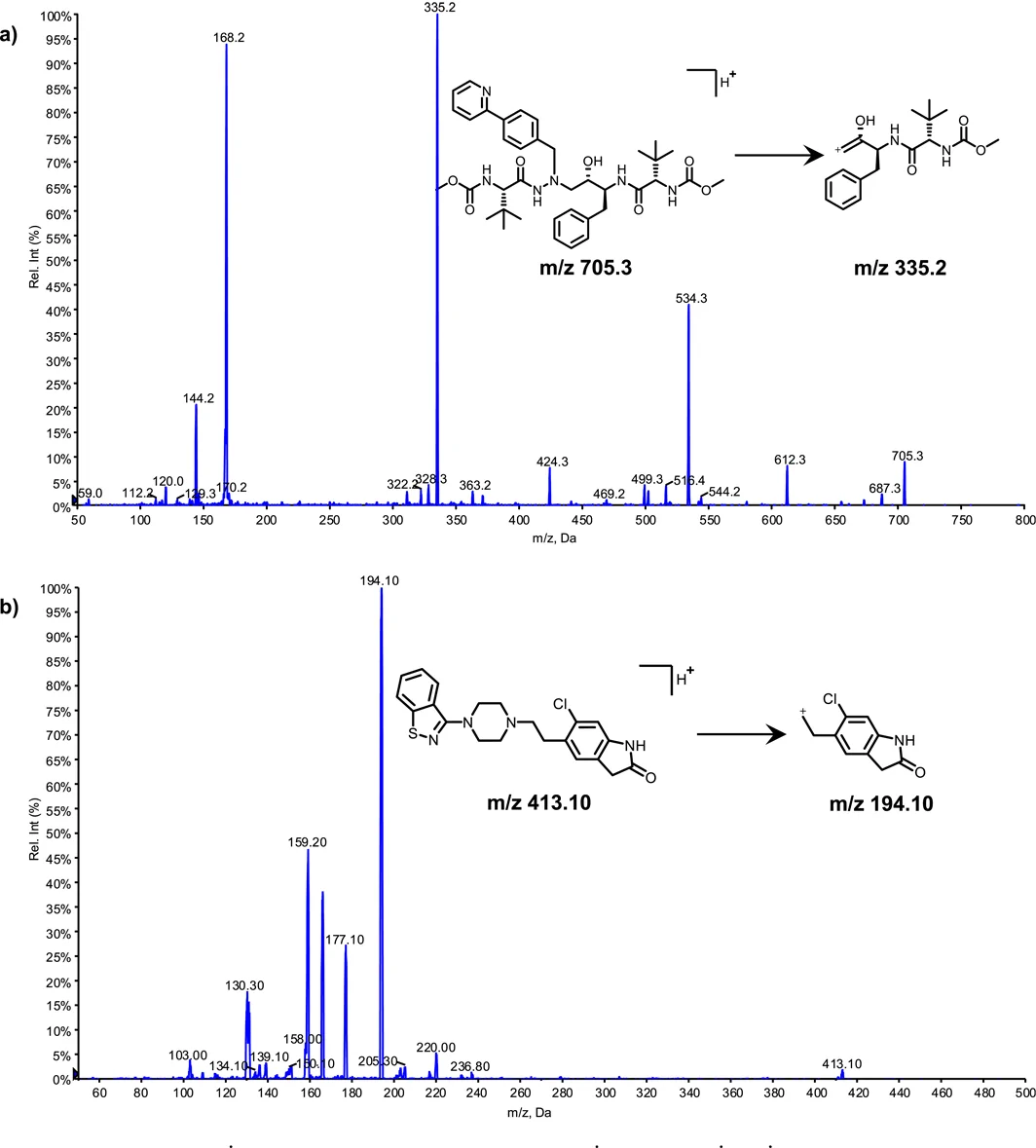
Product ion spectra of protonated atazanavir (a) and ziprasidone (b). The spectra confirm the major fragment ions used for mass spectrometric identification and quantification, with m/z 705.3 → 335.2 for atazanavir and m/z 413.1 → 194.1 for ziprasidone.

**FIGURE 2:**
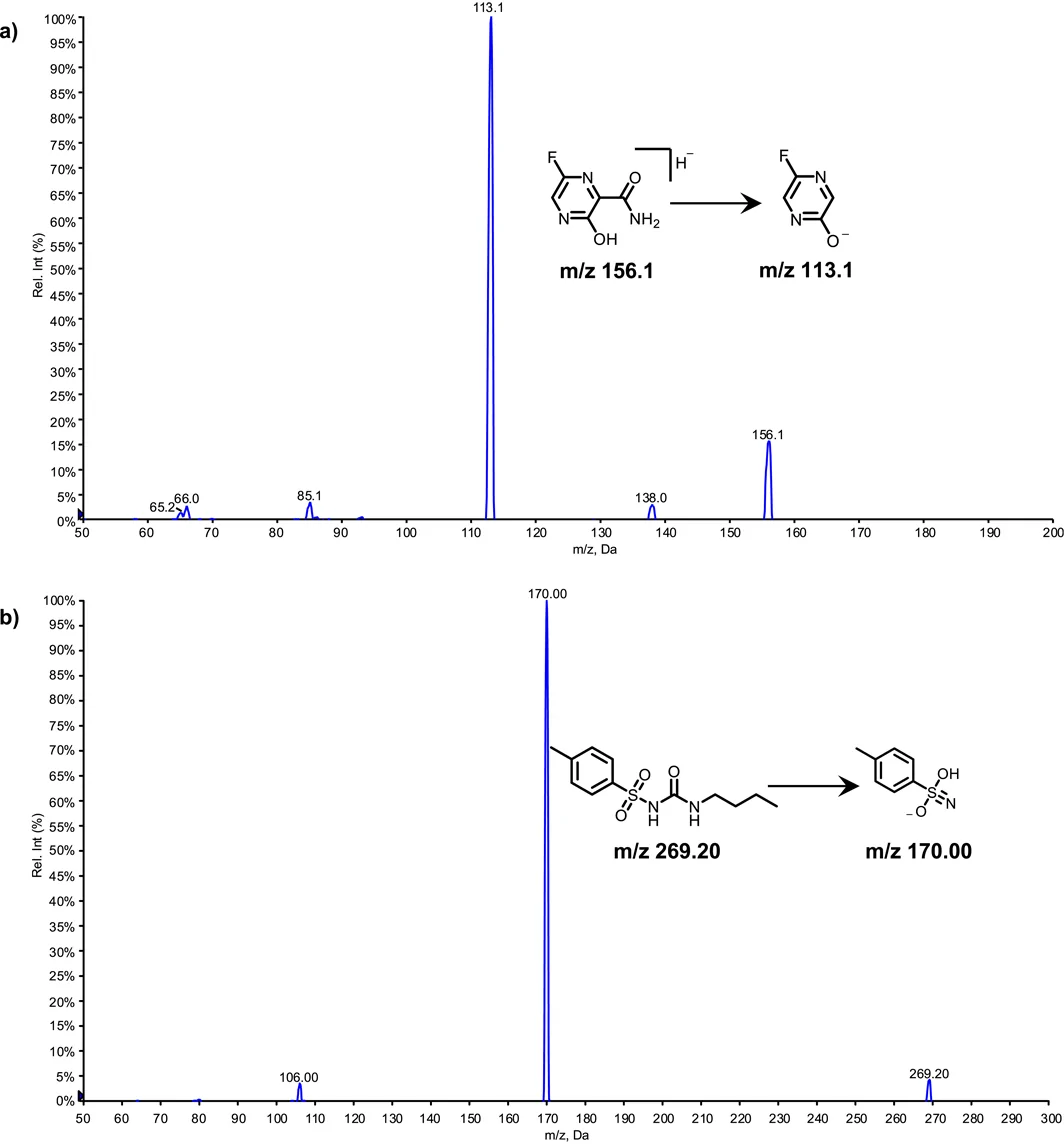
Product ion spectra of protonated favipiravir (a) and tolbutamide (b). The spectra confirm the major fragment ions used for detection in the LC–MS/MS method, with m/z 156.1 → 113.1 for favipiravir and m/z 269.2 → 170.0 for tolbutamide (internal standard).

Developing a robust chromatographic system is vital for the success of any bioanalytical method. The primary aim is to achieve efficient separation of analytes from endogenous substances and possible drug‐related byproducts. Co‐elution of these substances may lead to ion suppression or enhancement, compromising the accuracy of quantification. The first step in optimizing the chromatographic segment of an LC–MS/MS method is to examine the retention behavior of the compounds, typically using a reversed phase C18 column under both low‐ and high‐pH conditions to determine optimal retention and separation. Atazanavir and favipiravir, being weak bases, are better retained under acidic conditions due to their increased ionization and hydrophilicity in the presence of acidic pH buffer solutions. As a result, the ionized forms exhibit shorter retention times than their unionized counterparts. Atazanavir and favipiravir readily dissolve in organic solvents like acetonitrile and methanol. During optimization, several buffer systems and organic modifiers were explored across a range of pH values. The best chromatographic performance was achieved with a mobile phase containing methanol and ammonium formate (pH 3.0) for atazanavir and with a mobile phase containing methanol and 0.1% formic acid (pH 2.7) for favipiravir. Under these conditions, the respective IS was well resolved from the analytes, with excellent peak shape and sensitivity. The optimized chromatographic parameters resulted in no significant interference from endogenous matrix components. Figure 3 shows the extracted ion chromatograms of the analytes and their respective IS. This highly selective and sensitive LC‐QTRAP/MS method, validated under the described conditions, is suitable for pharmacokinetic studies and preclinical applications. It provides a reliable platform for quantifying atazanavir and favipiravir in plasma, supporting drug interaction and bioavailability studies in rat models.

**FIGURE 3:**
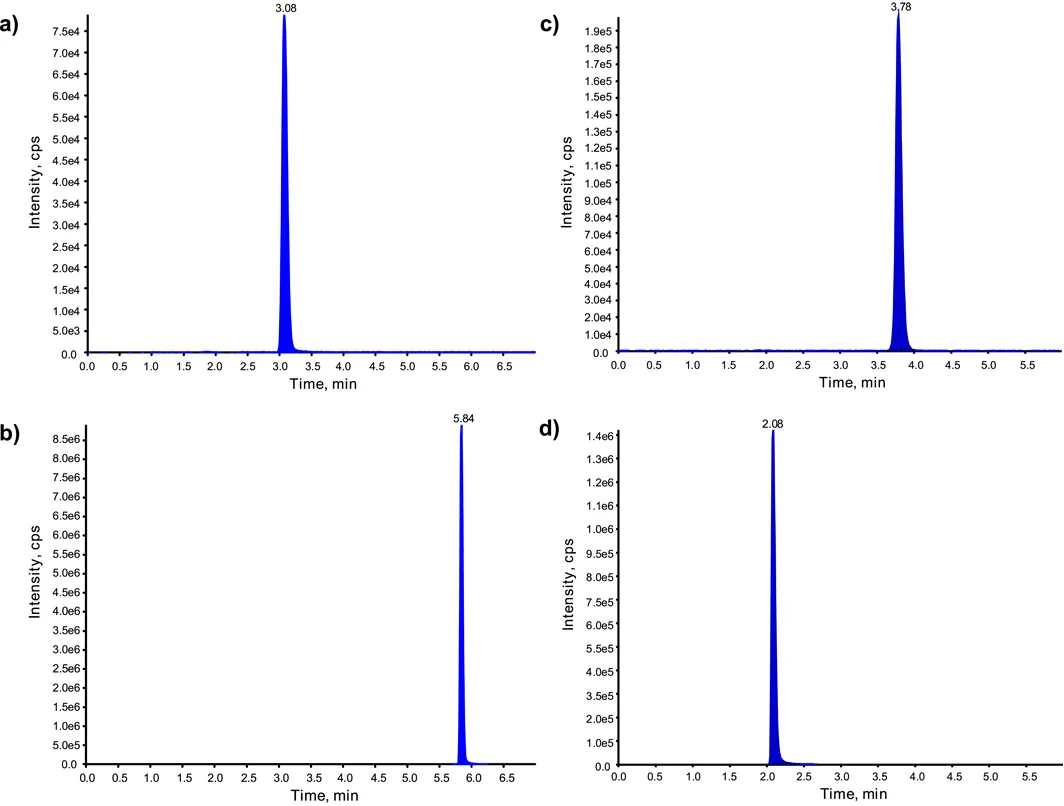
Extracted ion chromatograms of (a) favipiravir (retention time: 3.08 min), (b) tolbutamide (IS, retention time: 5.84 min), (c) atazanavir (retention time: 3.78 min), and (d) ziprasidone (IS, retention time: 2.08 min). The chromatograms confirm selective retention times and absence of significant interference at analyte peaks, supporting the reliability of the LCMS/MS method.

### 3.2 Method Validation

#### 3.2.1 Selectivity and Specificity

The selectivity of the established method was confirmed by verifying that no interfering peaks from endogenous substances in rat plasma were observed at the retention times of atazanavir and favipiravir, and their respective IS. The method aligns with FDA guidelines, enabling accurate identification and quantification of atazanavir and favipiravir in biological matrices. To assess carryover, blank samples were analyzed after the upper limit of quantification (ULOQ) samples. The results showed that carryover levels remained below the accepted threshold, ensuring precise results and minimizing contamination from previous samples.

#### 3.2.2 Recovery and Matrix Effect

The efficiency and consistency of the sample extraction method were evaluated through recovery and matrix effect studies. Table 2 outlines the percentage recoveries and matrix effects for three different quality control (QC) concentrations. The mean recovery rates for atazanavir and favipiravir were 83.80 ± 3.12 and 94.89 ± 1.22%, respectively, indicating that the extraction process was both effective and reproducible. Matrix effects were calculated at −2.72 ± 2.38% for atazanavir and −3.61 ± 1.72% for favipiravir, indicating slight ion suppression. Despite this, the observed matrix effects fell within the ± 15% threshold, confirming that they did not significantly compromise the method's accuracy or reliability. Internal standard–normalized matrix factors for ziprasidone (atazanavir) and tolbutamide (favipiravir) showed CV values below 10%, confirming adequate compensation for ion suppression or enhancement. The use of chilled methanol (−20°C) at a 1:3 plasma‐to‐solvent ratio yielded high recovery rates and clean extracts for both analytes.

**TABLE 2:** Recovery and matrix effect of atazanavir and favipiravir in rat plasma (*n* = 6, mean ± SD) at low (LQC), medium (MQC), and high (HQC) quality control levels.

Level	Atazanavir		Favipiravir	
	% of recovery	% of matrix effect	% of recovery	% of matrix effect
LQC	86.72 ± 2.28	−3.45 ± 1.8	95.09 ± 1.14	−3.38 ± 1.84
MQC	81.08 ± 3.17	−3.14 ± 2.16	94.55 ± 1.26	−3.67 ± 1.68
HQC	83.60 ± 3.92	−1.59 ± 3.20	95.04 ± 1.27	−3.78 ± 1.64

#### 3.2.3 Linearity and Sensitivity

Calibration curves for atazanavir and favipiravir demonstrated excellent linearity across the range of 10–2000 ng/mL in rat plasma. Atazanavir showed a coefficient of determination (*r* ^2^) of 0.998 with a regression equation of 0.0014x + 0.002, whereas favipiravir had an *r* ^2^ of 0.998 with a regression equation of *y* = 0.00017x – 0.000513. These values confirm a strong linear correlation between drug concentration and detector response. The back‐calculated concentrations from the calibration curve were within the accepted accuracy limits. At the lower limit of quantification (LLOQ), the signal‐to‐noise ratios met the sensitivity criteria outlined in the FDA guidance.

#### 3.2.4 Precision and Accuracy

Both intraday and inter‐day accuracy and precision assessments confirmed the reliability of the developed method for quantifying atazanavir and favipiravir in rat plasma. Table 3 summarizes the results, all of which comply with the FDA's recommended acceptance limits, demonstrating the method's reproducibility and consistency.

**TABLE 3:** Intra‐ and inter‐day precision and accuracy of atazanavir and favipiravir quality control samples in rat plasma (*n* = 6 and mean ± SD) at lower limit of quantification (LLOQ) and low (LQC), medium (MQC), and high (HQC) quality control levels.

Parameter	Atazanavir				Favipiravir			
	LLOQ	LQC	MQC	HQC	LLOQ	LQC	MQC	HQC
Intraday								
Mean ± SD	9.43 ± 0.90	31.22 ± 1.59	442.90 ± 18.93	739.04 ± 739.04	9.61 ± 0.14	29.02 ± 0.38	773.78 ± 12.64	1545 ± 27.51
%RSD	8.32	4.89	4.26	7.38	1.48	1.33	1.63	1.78
% of accuracy	94.35	104.07	110.72	92.38	96.14	96.74	96.72	96.59
Inter‐day								
Mean ± SD	9.79 ± 1.06	33.48 ± 1.10	440.10 ± 9.63	780.69 ± 64.07	9.66 ± 0.15	28.92 ± 0.54	779.60 ± 13.17	1552.08 ± 31.94
%RSD	9.47	3.16	2.18	8.19	1.53	1.86	1.69	2.06
% of accuracy	97.95	111.63	110.02	97.58	96.64	96.42	97.45	97.01

#### 3.2.5 Stability

The stability of atazanavir and favipiravir in rat plasma was evaluated under various storage and processing conditions using low and high QC samples (LQC and HQC). Stability was assessed by comparing stored samples with freshly prepared controls. The assay response variation did not exceed 5.67% for atazanavir and 5.02% for favipiravir, indicating minimal degradation. These findings, summarized in Table 4, suggest that both analytes remained stable throughout the tested conditions. Overall, the method proved robust and met all regulatory standards for routine pharmacokinetic analysis.

**TABLE 4:** Stability of atazanavir and favipiravir in rat plasma under various storage conditions, such as short‐term, long‐term, freeze–thaw, and postpreparative conditions (*n* = 6, mean ± SD) at (LQC) and high (HQC) quality control levels.

Stability condition	Spiked plasma concentration level	Atazanavir			Favipiravir		
		Measured concentration (ng/mL) (Mean ± SD)	% of RSD	% of stability	Measured concentration (ng/mL) (Mean ± SD)	% of RSD	% of stability
Room temperature stability for 6 h	LQC	2.93 ± 0.24	8.19	97.67	28.62 ± 0.66	2.31	95.40
	HQC	1592.84 ± 22.62	1.42	99.55	1523.07 ± 23.91	1.57	95.19
Three freeze–thaw cycles at −80°C	LQC	3.11 ± 0.20	6.43	103.67	28.90 ± 0.55	1.90	96.34
	HQC	1600.53 ± 14.73	0.92	100.03	1519.69 ± 39.06	2.57	94.98
Autosampler stability 5°C for 24 h	LQC	2.87 ± 0.06	2.09	95.67	29.07 ± 0.32	1.10	96.91
	HQC	1581.85 ± 28.98	1.83	98.86	1535.83 ± 28.26	1.84	95.98
Long‐term stability for 28 days at −80°C	LQC	3.17 ± 0.38	11.98	105.67	28.87 ± 0.86	2.98	96.24
	HQC	1592.16 ± 15.62	0.98	99.51	1533.31 ± 32.66	2.13	95.83

#### 3.2.6 Dilution Integrity and Incurred Sample Reanalysis

Dilution integrity at 20‐fold dilution showed accuracy within 93%–107% and precision (CV) below 10% for both analytes. ISR demonstrated that more than 90% of reassayed samples were within ± 20% of the original values, confirming analytical reliability.

### 3.3 Pharmacokinetic Evaluation of Herb–Drug Interaction

The present study investigated potential pharmacokinetic interactions between a commercial *Echinacea* supplement and the antiviral drugs favipiravir and atazanavir in rats. Two separate studies evaluated key pharmacokinetic parameters following oral administration of each drug alone or in combination with *Echinacea*. For favipiravir (Figure 4, Table 5), co‐administration with *Echinacea* caused a slight reduction in *C*_max_ (39,677 ± 5385 to 36,364 ± 5378 ng/mL) and a modest increase in *T*_max_ (0.67 ± 0.29 to 1.00 ± 0.00 h); however, these differences were not statistically significant and unlikely to be clinically relevant. Similarly, elimination parameters (elimination rate constant (K), elimination half‐life (*t*_1/2_)), systemic exposure measured by AUC_0–t_ and AUC_0–∞_, and mean residence time (MRT) showed small numerical changes without statistical significance, indicating that *Echinacea* did not significantly affect favipiravir's absorption, metabolism, or elimination.

**FIGURE 4:**
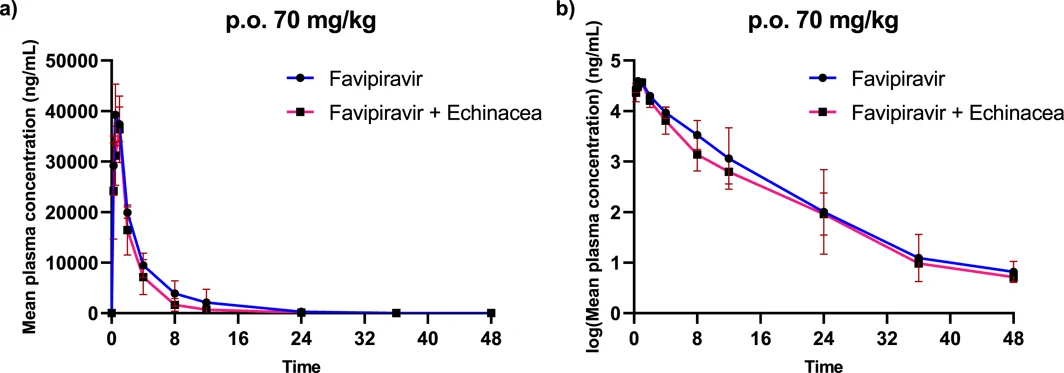
Mean plasma concentration‐time profiles of favipiravir in rats following repeated oral administration (70 mg/kg, p.o.) for 10 days, alone, and in combination with *Echinacea* (250 mg/kg, p.o.). Panel (a) shows data on a nonlogarithmic scale, whereas Panel (b) presents the same profiles on a logarithmic scale. Co‐administration with *Echinacea* produced modest changes in exposure without significant differences compared to favipiravir alone.

**TABLE 5:** Pharmacokinetics parameters of favipiravir after peroral administration of favipiravir and favipiravir in combination with *Echinacea* in SD rats (*n* = 6, mean ± SD).

Parameter	Favipiravir (70 mg/kg)	Favipiravir (70 mg/kg) + *Echinacea* (250 mg/kg)	*p*‐value
*C*_max_ (ng/mL)	39677.23 ± 5385.04	36364.00 ± 5378.50	0.493
*T*_max_ (h)	0.67 ± 0.29	1.00 ± 0.00	0.120
Rate constant (h^−1^)	0.19 ± 0.02	0.17 ± 0.02	0.288
*t*_1/2_ (h)	3.67 ± 0.41	4.10 ± 0.53	0.329
AUC_(0–*t*)_ (ng/mL*h)	144457.12 ± 17267.29	104840.21 ± 27001.20	0.099
AUC_(0–∞)_ (ng/mL*h)	144495.42 ± 17272.91	104870.65 ± 27002.81	0.099
MRT_(0–*t*)_ (h)	4.26 ± 1.83	3.13 ± 0.12	0.346
Vz/F (mL/kg)	1298.73 ± 256.30	2197.5 ± 911	0.175
Cl/F (mL/h/kg)	244.63 ± 30.28	360 ± 102	0.134

Abbreviations: AUC_(0–*t*)_: area under the concentration‐time curve from dosing (time 0) to the time of the last measured concentration; AUC_(0–∞)_: total area under the concentration‐time curve from dosing (time 0) taken to the limit; *C*_max_: maximum plasma concentration; *t*_1/2_: apparent terminal elimination half‐life; CL/F: apparent oral clearance; MRT_(0–*t*)_: mean residence time from dosing (time 0) to the time of the last measured concentration; *T*_max_: time of maximum plasma concentration; Vz/F: apparent volume of distribution during terminal phase.

Although minor changes in favipiravir pharmacokinetics were observed upon *Echinacea* co‐administration, none of the parameters demonstrated statistical significance. These findings suggest that under the conditions tested, *Echinacea* does not significantly alter the pharmacokinetic profile of favipiravir, making their concurrent use relatively safe in preclinical models, at least from a pharmacokinetic standpoint. However, it is essential to acknowledge that although statistical significance was not achieved, numerical trends were observed. These trends, though not definitive, warrant further consideration. It is possible that with a larger sample size, different *Echinacea* dosing regimens or favipiravir dosing regimens, or a different *Echinacea* formulation, or a statistically significant interaction might be observed. The observed trends could also indicate a subtle interaction that is not readily detectable in this study design. The potential mechanisms initially considered, such as inhibition of absorption, induction of efflux transporters, or enhanced metabolism, may still be relevant; however, their effects, if present, were not of sufficient magnitude to produce statistically significant changes in favipiravir pharmacokinetics in this study.

Atazanavir pharmacokinetics (Figure 5, Table 6) showed a slight increase in *C*_max_ upon *Echinacea* co‐administration (3274 ± 256 to 4004 ± 629 ng/mL), but no significant changes in *T*_max_, K, *t*_1/2_, or AUC measures were observed. The decrease in MRT appeared substantial but was not statistically significant, again suggesting biological variability rather than a drug interaction. There were no statistically significant alterations observed in any of the key pharmacokinetic parameters of atazanavir when co‐administered with *Echinacea*. This supports the assertion that at the administered doses and exposure durations, *Echinacea* does not exert a clinically meaningful impact on atazanavir pharmacokinetics. As with favipiravir, it is important to acknowledge the numerical trends. The initially considered mechanisms, such as inhibition of efflux transporters or CYP3A4 metabolism, may still be operative; however, their effects were not strong enough to produce statistically significant pharmacokinetic changes in this study. Atazanavir is a CYP3A4 and P‐glycoprotein substrate, while favipiravir is mainly cleared by aldehyde oxidase. *Echinacea* has been reported to modulate these pathways in a context‐dependent manner. In the present study, the absence of significant changes in AUC, *C*_max_, CL/F, or t_½_ indicates that *Echinacea* did not exert a functionally relevant effect on drug absorption, metabolism, or transport in vivo. The absence of such changes suggests that under the dosing conditions used, *Echinacea* does not alter the rate or extent of absorption of either drug, nor does it significantly influence hepatic or intestinal clearance mechanisms. While subtle molecular changes in enzyme or transporter expression cannot be ruled out, the present pharmacokinetic data demonstrate that these do not translate into altered systemic drug exposure, which is the key determinant of clinical relevance.

**FIGURE 5:**
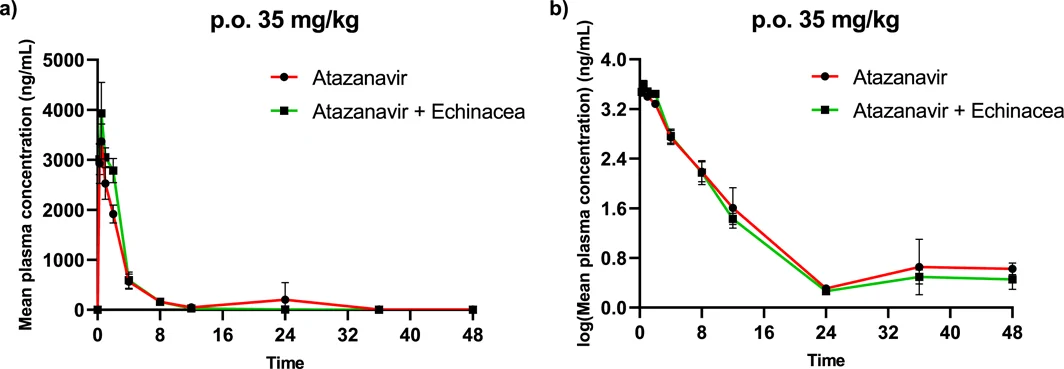
Mean plasma concentration‐time profiles of atazanavir in rats following repeated oral administration (30 mg/kg, p.o.) for 10 days, alone, and in combination with *Echinacea* (250 mg/kg, p.o.). Panel (a) shows data on a nonlogarithmic scale, whereas panel (b) presents the same profiles on a logarithmic scale. Co‐administration with *Echinacea* produced minor changes in systemic exposure, but overall pharmacokinetic profiles remained comparable to atazanavir alone.

**TABLE 6:** Pharmacokinetics parameters of atazanavir after peroral administration of favipiravir and atazanavir in combination with *Echinacea* in SD rats (*n* = 6, mean ± SD).

Parameter	Atazanavir (70 mg/kg)	Atazanavir (70 mg/kg) + *Echinacea* (250 mg/kg)	*p*‐value
*C*_max_ (ng/mL)	3274.32 ± 256.40	4003.59 ± 628.64	0.136
*T*_max_ (h)	0.50 ± 0.00	0.54 ± 0.22	0.769
Rate constant (h^−1^)	0.15 ± 0.01	0.15 ± 0.01	1.000
*t*_1/2_ (h)	4.62 ± 0.16	4.50 ± 0.24	0.511
AUC_(0–*t*)_ (ng/mL*h)	8962.17 ± 1667.35	10644.55 ± 2948.53	0.438
AUC_(0–∞)_ (ng/mL*h)	8990.58 ± 1669.48	10664.13 ± 2948.24	0.441
MRT_(0–*t*)_ (h)	2.84 ± 0.43	2.49 ± 0.47	0.395
Vz/F (mL/kg)	27011 ± 5639	206323 ± 265410.1	0.307
Cl/F (mL/h/kg)	4036 ± 771	31341 ± 39710.1	0.300

Abbreviations: AUC_(0–*t*)_: area under the concentration‐time curve from dosing (time 0) to the time of the last measured concentration; AUC_(0–∞)_: total area under the concentration‐time curve from dosing (time 0) taken to the limit; *C*_max_: maximum plasma concentration; *T*_max_: time of maximum plasma concentration; CL/F: apparent oral clearance; MRT_(0–*t*)_: mean residence time from dosing (time 0) to the time of the last measured concentration; *t*_1/2_: apparent terminal elimination half‐life; Vz/F: apparent volume of distribution during terminal phase.

Contrary to our initial hypothesis, these two herb–drug interaction studies, focused on favipiravir and atazanavir co‐administered with *Echinacea*, demonstrate no statistically significant changes in pharmacokinetic parameters for either drug in rat models. This suggests that at least within the limits of the study design, *Echinacea* does not significantly influence the systemic exposure, absorption kinetics, or elimination profiles of either favipiravir or atazanavir. From a mechanistic perspective, while *Echinacea* has been shown in other contexts to modulate the activity of cytochrome P450 enzymes (notably CYP3A4), uridine diphosphate glucuronosyltransferases (UGTs), and efflux transporters (such as P‐gp), its effects are often dose‐dependent, species‐specific, and influenced by the duration of administration. Previous studies evaluating *Echinacea* interactions with CYP3A4 and P‐gp substrates have reported variable outcomes, ranging from mild inhibition to modest induction, depending on extract composition and exposure duration. Clinical and preclinical investigations involving substrates such as midazolam, cyclosporine, digoxin, and HIV protease inhibitors have demonstrated limited or no clinically relevant pharmacokinetic interactions with *Echinacea* preparations (Gurley et al. 2004, 2008; Penzak et al. 2010; Moltó et al. 2012; Woelkart et al. 2008). The absence of meaningful changes in favipiravir and atazanavir exposure observed in this study aligns with these reports, supporting the conclusion that *Echinacea* typically exerts weak or clinically insignificant modulation of major drug‐metabolizing enzymes and transporters when administered as a commercial supplement. Clinical and preclinical studies with drugs such as midazolam, cyclosporine, and digoxin have generally shown either no effect or only minor, transient changes in systemic exposure (Freeman and Spelman 2008; Gurley et al. 2004; Moltó et al. 2011; Penzak et al. 2010). The absence of meaningful changes in atazanavir and favipiravir pharmacokinetics in the present study is consistent with these reports and supports the conclusion that *Echinacea* typically exerts weak or clinically insignificant modulation of major drug‐metabolizing enzymes and transporters when administered as a commercial supplement. Post hoc power analysis using the observed variability in Cmax and AUC (Table 7) confirmed that the present study had 80%–90% power to detect ≥ 25% changes in systemic exposure, which is the regulatory threshold for a clinically meaningful pharmacokinetic interaction. Therefore, the absence of statistically significant differences is unlikely to be due to an insufficient sample size and instead supports the conclusion that *Echinacea* does not meaningfully alter the exposure to favipiravir or atazanavir. Although minor numerical differences were observed in some pharmacokinetic parameters, these changes did not reach statistical significance. The absence of statistically significant differences indicates that *Echinacea* did not produce a pharmacokinetically relevant interaction with favipiravir or atazanavir under the conditions investigated. Accordingly, these numerical trends should be interpreted with caution and not overemphasized in the absence of statistical and exposure‐based relevance. Although quantitative differences in drug disposition exist across species, rat pharmacokinetic interaction studies are widely used as an initial approach to identifying potential herb–drug interaction risks in humans. Atazanavir and favipiravir share qualitatively similar disposition pathways in rats and humans, particularly with respect to CYP3A‐mediated atazanavir metabolism. Therefore, the absence of meaningful changes in systemic exposure observed in this repeated‐dosing rat model suggests a low likelihood of clinically relevant pharmacokinetic interactions in humans. From a translational perspective, these findings support further targeted clinical evaluation, such as single‐dose or steady‐state pharmacokinetic studies in healthy volunteers using standardized *Echinacea* preparations, if warranted. Although molecular‐level mechanistic data were not generated, the present study provides exposure‐based evidence that repeated co‐administration of *Echinacea* does not result in a pharmacokinetically relevant interaction with favipiravir or atazanavir. Because systemic pharmacokinetic parameters reflect the net functional consequences of enzyme and transporter modulation, the absence of significant exposure changes indicates that any underlying molecular effects, if present, are unlikely to be clinically meaningful under the conditions studied. The data suggest that short‐term co‐administration of *Echinacea* with favipiravir or atazanavir may not necessitate dose adjustment or therapeutic drug monitoring, although confirmation in human studies is essential. Moreover, the results emphasize the importance of conducting well‐controlled preclinical herb–drug interaction studies using validated LC–MS/MS bioanalytical methods, as subtle, nonsignificant numerical trends may still provide valuable preliminary insights to guide clinical decision‐making and further research. A limitation of this study is that the *Echinacea* supplement was not independently and phytochemically characterized beyond manufacturer certification. However, a commercially standardized product was deliberately selected to reflect typical patient use. Importantly, the absence of meaningful changes in systemic drug exposure indicates that regardless of phytochemical variability, the preparation did not produce a pharmacokinetically relevant interaction under the investigated conditions.

**TABLE 7:** Post hoc power analysis for detection of pharmacokinetic interaction.

Drug	Parameter	CV (%)	Effect size tested	Power (*n* = 6 per group)
Favipiravir	*C*_max_	13–15	25%	88%
Favipiravir	AUC_(0–∞)_	12–26	25%	82%
Atazanavir	*C*_max_	8–16	25%	90%
Atazanavir	AUC_(0–∞)_	19–28	25%	80%

## 4 Conclusion

In this study, we successfully developed and validated robust LC‐QTRAP/MS methods for the quantitative analysis of favipiravir and atazanavir in rat plasma. Both methods demonstrated excellent performance characteristics, including high sensitivity, selectivity, precision, accuracy, and linearity, and serve as reliable and efficient tools for quantifying both drugs. The developed methods were applied to assess the herb–drug interactions. The co‐administration of a commercially available *Echinacea* supplement did not result in statistically significant alterations in the pharmacokinetic profiles of either favipiravir or atazanavir in rats, under the conditions of this experiment. Although numerical trends were observed, these did not reach statistical significance. These findings suggest that, at the dose and under the conditions of this study, *Echinacea* is unlikely to have a clinically relevant pharmacokinetic interaction with these two antiviral drugs in rats. Nevertheless, given the variability in herbal supplement composition, patient metabolism, and treatment duration in clinical settings, further studies, particularly human clinical trials, are warranted to corroborate these findings and fully assess safety.

## Funding

The authors have nothing to report.

## Conflicts of Interest

The authors declare no conflicts of interest.

## Data Availability

The data that support the findings of this study are available from the corresponding author upon reasonable request.
